# Supplementary material for: Association between attending exercise-based cardiac rehabilitation and cardiovascular risk factors at one-year post myocardial infarction
Source: PLoS One. 2020 May 11;15(5):e0232772. doi: 10.1371/journal.pone.0232772 (PMC7213725; doi:10.1371/journal.pone.0232772)
Supplement: Attachment — Submitted filename: Response to reviewers.docx [file pone.0232772.s001.docx]

**PONE-D-19-32783**
Association between attending exercise-based cardiac rehabilitation and cardiovascular risk factors at one-year post myocardial infarction
PLOS ONE

Dear Dr Leosdottir,
Thank you for submitting your manuscript to PLOS ONE. After careful consideration, we feel that it has merit but does not fully meet PLOS ONE’s publication criteria as it currently stands. Therefore, we invite you to submit a revised version of the manuscript that addresses the points raised during the review process.

*Please address comments from all reviewers.*

We would appreciate receiving your revised manuscript by Jan 31 2020 11:59PM. When you are ready to submit your revision, log on to https://www.editorialmanager.com/pone/ and select the 'Submissions Needing Revision' folder to locate your manuscript file.

If you would like to make changes to your financial disclosure, please include your updated statement in your cover letter.

To enhance the reproducibility of your results, we recommend that if applicable you deposit your laboratory protocols in protocols.io, where a protocol can be assigned its own identifier (DOI) such that it can be cited independently in the future. For instructions see: <http://journals.plos.org/plosone/s/submission-guidelines#loc-laboratory-protocols>

Please include the following items when submitting your revised manuscript:

- A rebuttal letter that responds to each point raised by the academic editor and reviewer(s). This letter should be uploaded as separate file and labeled 'Response to Reviewers'.
- A marked-up copy of your manuscript that highlights changes made to the original version. This file should be uploaded as separate file and labeled 'Revised Manuscript with Track Changes'.
- An unmarked version of your revised paper without tracked changes. This file should be uploaded as separate file and labeled 'Manuscript'.

Please note while forming your response, if your article is accepted, you may have the opportunity to make the peer review history publicly available. The record will include editor decision letters (with reviews) and your responses to reviewer comments. If eligible, we will contact you to opt in or out.

We look forward to receiving your revised manuscript.

Kind regards,

Gordon McGregor
Academic Editor
PLOS ONE

## Journal Requirements

When submitting your revision, we need you to address these additional requirements.

Please ensure that your manuscript meets PLOS ONE's style requirements, including those for file naming. The PLOS ONE style templates can be found at

<http://www.journals.plos.org/plosone/s/file?id=wjVg/PLOSOne_formatting_sample_main_body.pdf> and <http://www.journals.plos.org/plosone/s/file?id=ba62/PLOSOne_formatting_sample_title_authors_affiliations.pdf>

***Author response:*** *We have double checked that the manuscript meets PLOS ONE´s style requirements.*

We note that you have indicated that data from this study are available upon request. PLOS only allows data to be available upon request if there are legal or ethical restrictions on sharing data publicly. For information on unacceptable data access restrictions, please see http://journals.plos.org/plosone/s/data-availability#loc-unacceptable-data-access-restrictions.

In your revised cover letter, please address the following prompts:

a) If there are ethical or legal restrictions on sharing a de-identified data set, please explain them in detail (e.g., data contain potentially identifying or sensitive patient information) and who has imposed them (e.g., an ethics committee). Please also provide contact information for a data access committee, ethics committee, or other institutional body to which data requests may be sent.

b) If there are no restrictions, please upload the minimal anonymized data set necessary to replicate your study findings as either Supporting Information files or to a stable, public repository and provide us with the relevant URLs, DOIs, or accession numbers. Please see http://www.bmj.com/content/340/bmj.c181.long for guidelines on how to de-identify and prepare clinical data for publication. For a list of acceptable repositories, please see http://journals.plos.org/plosone/s/data-availability#loc-recommended-repositories.

We will update your Data Availability statement on your behalf to reflect the information you provide.

***Author response:*** *The data used in this study is from the SWEDEHEART registry. Access to data from the registry needs to be applied for and third-party data usage is not allowed, irrespective of whether the data contain potentially identifying or sensitive data or not. Instead, given ethical study approval from the Swedish Ethical Review Authority, access to SWEDEHEART data supporting the present findings can be applied for from the Uppsala Clinical Research Center (UCR) in Sweden. Further information can be found on the UCR* [*https://www.ucr.uu.se/en/*](https://www.ucr.uu.se/en/) *and Swedish Ethical Review Authority* [*https://etikprovningsmyndigheten.se/*](https://etikprovningsmyndigheten.se/) *websites.*

Reviewers' comments

Reviewer's Responses to Questions

**Comments to the Author**

1. Is the manuscript technically sound, and do the data support the conclusions?

The manuscript must describe a technically sound piece of scientific research with data that supports the conclusions. Experiments must have been conducted rigorously, with appropriate controls, replication, and sample sizes. The conclusions must be drawn appropriately based on the data presented.

Reviewer #1: Yes

Reviewer #2: Yes

Reviewer #3: Yes

Reviewer #4: Yes

2. Has the statistical analysis been performed appropriately and rigorously?

Reviewer #1: Yes

Reviewer #2: Yes

Reviewer #3: I Don't Know

Reviewer #4: Yes

3. Have the authors made all data underlying the findings in their manuscript fully available?

The [PLOS Data policy](http://www.plosone.org/static/policies.action#sharing) requires authors to make all data underlying the findings described in their manuscript fully available without restriction, with rare exception (please refer to the Data Availability Statement in the manuscript PDF file). The data should be provided as part of the manuscript or its supporting information, or deposited to a public repository. For example, in addition to summary statistics, the data points behind means, medians and variance measures should be available. If there are restrictions on publicly sharing data—e.g. participant privacy or use of data from a third party—those must be specified.

Reviewer #1: Yes

Reviewer #2: Yes

Reviewer #3: Yes

Reviewer #4: Yes

4. Is the manuscript presented in an intelligible fashion and written in standard English?

PLOS ONE does not copyedit accepted manuscripts, so the language in submitted articles must be clear, correct, and unambiguous. Any typographical or grammatical errors should be corrected at revision, so please note any specific errors here.

Reviewer #1: Yes

Reviewer #2: Yes

Reviewer #3: Yes

Reviewer #4: Yes

6. PLOS authors have the option to publish the peer review history of their article ([what does this mean?](https://journals.plos.org/plosone/s/editorial-and-peer-review-process#loc-peer-review-history)). If published, this will include your full peer review and any attached files. If you choose “no”, your identity will remain anonymous but your review may still be made public.

**Do you want your identity to be public for this peer review?** For information about this choice, including consent withdrawal, please see our [Privacy Policy](https://www.plos.org/privacy-policy).

Reviewer #1: No

Reviewer #2: No

Reviewer #3: No

Reviewer #4: No

[NOTE: If reviewer comments were submitted as an attachment file, they will be attached to this email and accessible via the submission site. Please log into your account, locate the manuscript record, and check for the action link "View Attachments". If this link does not appear, there are no attachment files to be viewed.]

5. Review Comments to the Author

Please use the space provided to explain your answers to the questions above. You may also include additional comments for the author, including concerns about dual publication, research ethics, or publication ethics. (Please upload your review as an attachment if it exceeds 20,000 characters)

## Reviewer #1

It is always good to see actual audit type data against which to compare data collected from studies. The paper is presented in a logical easy to read and clear format, though a number of considerations are required to get it to publishable standard. Generally the Discussion was descriptive and needs more intra and inter-study critical analyses and comparisons.

How do the data compare with the UK's NACR and the US AACVPR data sets/registries and other registries from Europe?

***Author response:*** *We thank the Reviewer for this important comment. While there is an abundance of registry-based studies on outcomes in CR in general, we have not been able to identify registry-based studies exploring associations between exercise training within CR in particular and CVD outcomes. This limitation concerning registry data in Europe has been recognized (EuroCaReD study group. Exercise-based cardiac rehabilitation in twelve European countries results of the European cardiac rehabilitation registry. Int J Cardiol. 2017 Feb 1; 228:58-67). If the Reviewer can point us to some useful references with registry-based data on exercise training within CR we would be more than happy to incorporate these into the manuscript.*

As this study has a strong focus on physical activity, in making comparisons with the evidence, a key element which makes the findings weak is that the outcomes have only been linked with frequency of physical activity. Do the SWEDEHeart programmes not measure fitness and exercise capacity? Aerobic fitness is a very strong risk factor; far stronger than physical activity. The discussion alludes to changes in aerobic fitness, so if you do not have these measures it is important to highlight this weakness and make recommendations for future Registry Outcomes. Is there in any information in the Data sets to whether or not patients actually performed the 20+ minutes at an RPE >13?

***Author response:*** *Unfortunately, measures of aerobic fitness were not registered in SWEDEHEART at the time of the study.* *Since 2018, however, new registry outcomes of aerobic capacity and muscle strength have been added, making it possible to measure changes in physical fitness in future studies.*

*Also, when the study was conducted, there was not yet a clear definition in the registry on how to define a successfully completed exCR programme. Even though the total duration of the programme was recommended to be at least three months, one could still answer yes to this variable even if the patient hadn´t attended a full three-month programme. Therefore, we cannot report on number of attended exercise sessions, and as such this could potentially vary considerably between patients.*

*In addition, we agree with the Reviewer that the PA level per se has not same clear effects on outcomes in patients with CAD, as compared to physical fitness. We have added these aspects in the revised version of the Discussion.*

Another key weakness which needs to be noted is the single use of Self-Reported Physical activity. Remarks in the Discussion should be made on the validity and reliability of this method. The final point on physical activity, is that although the differences between attenders and non-attenders participation levels were statistically significant (presumably due to the large sample size), is a 0.5 day difference actually clinically meaningful? This small real difference, though statistically significant, could likely be a function the limitations of Self Report, which are known to greatly over-report in Physical Activity (see reports from S. Prince from Statistics Canada) Again, the benefit of this difference would have been illuminated if exercise capacity were measured as a risk factor outcome.

***Author response:*** *We agree with this comment and have added text on limitations of self-reports in the revised version of the Discussion.*

In terms of manuscript presentation clarity and noting the main results are summarised in Tables 4 and 5, the titles of these tables seem a little misleading and also need more precision. The term "association" would lead one to believe a correlation statistic was being used and not a test of "difference" being assessed. I would recommend that these tables are renamed with such recommended words as; Differences in CVD risk factors 12-months after treatment of a CHD event, in those who attended versus those who declined cardiac rehabilitation. We cannot rule out a selection bias whereby potentially the most motivated patients have chosen to participate in exCR. This must be further investigated in studies using RCT design.

***Author response:*** *We thank the Reviewer for pointing this out.* *The title of the table (now joint for both sexes in accordance to comment from Reviewer #3) has been amended, as well as text in the manuscript referring to the results presented in the table.*

## Reviewer #2

This study appears to be methodologically sound, and generates interesting results, particularly around the gender differences. The only critique that I have is around the fact that there is no data around adherence to / % completion of the programme - I do think that would be interesting to know, especially given that women have a higher attendance than men (which is in contrast to what's found typically in other countries).

***Author response:*** *We agree that this is a major limitation to the analysis, and this is highlighted in the Strengths and weaknesses section of the Discussion. Unfortunately, at the time of the study, there was no detailed registration of the number of attended exCR programme sessions in SWEDEHEART. The question was formulated “Has the patient attended a structured exCR programme post MI?” (self-reported by the patient or documented by a physiotherapist in the patient´s medical records), and was registered at one-year follow-up. If yes was selected, there was a further selection of whether the patient attended the programme for <3 months or ≥3 months, as the latter is what is recommended by SWEDEHEART. As the majority of both men and women attended exCR for <3 months (see table below) we pooled the two answers (<3 months or ≥3 months) together.*

*We did perform a secondary analysis, not included in the manuscript, on subgroups according to attendance (<3 months or ≥3 months) and found no major differences in outcomes. As the results did not differ from the main/pooled results and to limit word count we did not include these secondary analysis results in the manuscript.*

*We can add that since 2018, the number of attended exCR sessions is registered in SWEDEHEART. Also, objective measures aerobic capacity and muscle strength have been introduced in the registry, with patients´ results from submaximal ergometer bicycle test and muscle strength tests before and after attending exCR are registered. In future analysis of SWEDEHEART registry data these more detailed measures will undoubtedly give new insights into the relevance of physical fitness and exCR attendance on patient outcomes from real-life settings.*

*The definition of an exCR programme according to SWEDEHEART is a supervised centre-based programme, led by a physiotherapist, containing both aerobic training and resistance training with a total session length of 60 minutes. Each exercise session should include at least 20 minutes of aerobic exercise (intensity 13-17 on Borg´s rating of perceived exertion (RPE) scale) and resistance exercise performed in 1-3 sets of 8-10 upper and lower limb exercises in 10-15 repetitions. The programme should include three sessions per week whereof one session can be conducted at a local healthcare facility or at home.*

|  | *Did not attend exCR* | *Attended exCR <3 months* | *Attended ≥exCR 3 months* |
| --- | --- | --- | --- |
| *Men* | *8161 (57%)* | *4381 (31%)* | *1770 (12%)* |
| *Women* | *2689 (56%)* | *1510 (31%)* | *625 (13%)* |

## Reviewer #3

The authors present an interesting report on effects of exercise-based CR on cardiovascular risk factors. The retrospectively analysed dataset is large and includes a non-randomised control group. The data is timely and provides valuable data that reports on the effects of cardiac rehabilitation in a ‘real world’ setting. The manuscript would benefit from some modifications and/or clarifications.

Abstract

Well written – The authors may wish to revise some of the conclusions based on my comments, although I will leave this for them to decide.

***Author response:*** *The abstract has been revised in accordance to changes made to the manuscript.*

Background
The background and rationale is well articulated.

Line 62 – I agree that the effect of exercise-based CR in the modern era of MI treatment is less well document. However, the systematic review and meta-analysis by Powell et al. (2018) investigated this topic in relation to mortality/morbidity and found no effect. Please could you briefly acknowledge the findings of ‘Is exercise-based cardiac rehabilitation effective? A systematic review and meta-analysis to re-examine the evidence’.

***Author response:*** *A sentence referring to Powell et al. (2018) has been added to the Background section.*

Methods

Line 101 – Could patients who don’t complete a full cardiac rehabilitation programme be included in this analysis? For example, could someone who undertakes 2 months of rehabilitation be recorded as someone who attended cardiac rehabilitation? (yes/no). Equally, is it likely that many patients had more than 3 months of cardiac rehabilitation, and therefore, had a greater treatment ‘dose’.

***Author response:*** *If the Reviewer is referring to the exCR programme, the answer is yes.* *Unfortunately, at the time of the study, there was no detailed registration of the number of attended exCR programme sessions in SWEDEHEART. The question was formulated “Has the patient attended a structured exCR programme post MI?” (self-reported by the patient or documented by a physiotherapist in the patient´s medical records), registered at one-year follow-up. If yes was selected, there was a further selection of whether the patient attended the programme for <3 months or ≥3 months, as the latter is what is recommended by SWEDEHEART.*

*We acknowledge that this is a major limitation to the analysis, as is highlighted in the Strengths and weaknesses section of the Discussion.*

Table 1 – Please clarify if lipids were measured from fasting/non-fasting blood samples.

***Author response:*** *In the SWEDEHEART user manual it is recommended that lipids are measured according to recommendations from the local lab. Whether they are measured fasting or non-fasting is not registered in the registry. This has been clarified in the revised manuscript.*

Line 121 – The use of CHD and CAD are both used in the manuscript. Please amend use one throughout the manuscript for consistency.

***Author response:*** *The manuscript has been revised and now only the term CAD is used.*

Line 139 – pleased change to ‘reported)’. Also, please amend to ‘otherwise it is self-reported).

***Author response:*** *These changes have been made, also in other parts of the manuscript (changing self-report to self-reported).*

143 – Are you able to say whether BP measurements are generally obtained from automated or sphygmomanometer and stethoscope?

***Author response:*** *Unfortunately, information on whether blood pressure is measured with and automated blood pressure monitor or with a sphygmomanometer and stethoscope is not included in the SWEDEHEART registry. The following sentence has been rephrased in the revised manuscript:*

*“*Generally, at Swedish CR centres a blood pressure measurement is performed with the patient in supine position after five minutes of rest, either with an automated blood pressure monitor or sphygmomanometer and stethoscope.”

Line 159 – This sentence is a little confusing. Please can you clarify that means and SD were used to report normally distributed data, and medians with IQR were used to report non-normally distributed data. Please can you also clarify how data normality were assessed. Please change ‘skewed ’to read ‘non-normally distributed’ or ‘data that was not normally distributed because assessing kurtosis is also part of determining normality.

***Author response:*** *Thank you for the comment. In our original analysis we only assessed normality by visual inspection of histograms. We have now assessed normality more thoroughly, by also calculating skewness and kurtosis. This has been added to the Statistical analysis section, and numbers in the Baseline characteristics table adjusted accordingly.*

Line 175 – Please can you justify why beta-blockers weren’t included as a covariate? They commonly have an effect on blood pressure as well as resting heart rate.

***Author response:*** *We thank the Reviewer for this valuable and important comment. The analyses have been performed again, adding the use of beta-blockers to the regression models. The new model resulted in the difference in systolic blood pressure and glucose for men losing significance. The results table has been updated and the text in the Results and Discussion sections, as well as the Abstract, have been amended in light of the changed results.*

General comment – It isn’t clear from the methods, why women and men were analysed separately. It would be useful to report differences between all four conditions; male attenders, male non-attenders, female attenders, and female non-attenders. The comments below relate to this point.

***Author response:*** *Justification for analysing men and women separately is given in the Introduction section of the manuscript: …*it is unclear whether attendance in exCR results in the same benefits in real-life settings as that observed in randomized trials, especially so in women, as several studies have indicated both uptake and outcomes in CR to be inferior among women. *Additionally, women have often been underrepresented in previous studies. Whether current evidence applies to men and women to the same degree, can as such be questioned. This has been added to the Introduction:*

However, many studies on exCR were performed before statins, angiotensin converting enzyme (ACE) inhibitors and percutaneous coronary interventions (PCI) became an integral part of MI treatment and often predominantly included male patients, limiting generalizability to both sexes [6, 7]. Whether the benefits of exCR on patient outcomes apply in the modern era of MI treatment and to men and women alike, is less well documented.

*In our opinion, the justification for choice of methods should be stated in the Introduction, in context with the aims of the study. However, if the Reviewer and Editor feel that the information is better suited in the Methods section, we can amend the manuscript accordingly.*

*The results tables have been joined for men and women (non-attenders and attenders), as the Reviewer suggests.*

Results

Table 2 – It would be helpful if the descriptive statistics for men and women were presented in one table.

***Author response:*** *Tables 2 and 3 have been joint into one table.*

Table 2 – Please explain what wet snuff (%) is. Many readers may not be familiar with this.

***Author response:*** *The term wet snuff has been exchanged by moist snuff (dipping tobacco), which are more commonly used terms.*

Line 212 – See comments above. Would it be more informative to compare males and females if these data were analysed as four different groups, and reported in one table?

***Author response:*** *Tables 4 and 5 have been joint into one table.*

215 – Please explain what is meant by being ‘abstinent’ from smoking. This isn’t defined in the methods. Smoking status is reported differently in Tables 4 and 5, compared with Tables 2 an 3. Until these variables are consistently reported, it makes the conclusions around the smoking status difficult to justify as the data could be misleading.

***Author response:*** *As stated in the Methods section, smoking was self-reported as being a current smoker, ex-smoker (stopped smoking >1 month) or never having smoked at baseline and at follow-up visits. The formulation in the manuscript has been harmonized, to avoid confusion. In the Results section, the sentence previously reading…:*

*“*For both men and women, attending exCR was significantly associated with a larger proportion being abstinent from smoking (current smokers at baseline only)…”

*…now reads:*

“For both men and women, those who attended exCR were significantly more often reported having stopped smoking after the MI (current smokers at baseline only)…”

Discussion

The claims surrounding the statistically significant reduction in risk factors is fine. However, some of the significant reductions are small e.g. 0.1 mmol/ greater reduction in triglycerides among women taking part in CR. What is the clinical value of this, and how does this compare to data from clinical trials? This would be valuable information to reader because the aim of the study is to examine whether exercise-based CR results in beneficial changes in cardiovascular risk factors in a ‘real life’ setting.

***Author response:*** *We agree that the small effect on triglycerides is probably of limited clinical significance and could be a result of mass-significance. The 0.3 mmol/l difference in LDL reduction between female attenders and non-attenders could on the other hand be of clinical significance. Based on the extensive meta-analyses from the CTT collaboration (Efficacy and safety of more intensive lowering of LDL cholesterol: a meta-analysis of data from 170,000 participants in 26 randomised trials. Lancet. 2010 Nov 13;376(9753):1670-81) a 1 mmol/L reduction in LDL results in a 21% reduction in mortality risk, which would translate to approximately 7% future mortality risk reduction for female attenders in our study.*

*The clinical value of changes in physical activity levels to the extent observed in our study, has been indicated in Ekblom et al (Increased Physical Activity Post-Myocardial Infarction Is Related to Reduced Mortality: Results From the SWEDEHEART Registry. J Am Heart Assoc. 2018 Dec 18;7(24):e010108. ) and Gorczyca AM, et al (Change in physical activity and sitting time after myocardial infarction and mortality among postmenopausal women in the Women's Health Initiative‐Observational Study. J Am Heart Assoc. 2017; 6:e005354.) In the Ekblom et al study, which is based on SWEDEHEART data using the same self-report instrument as in our study, those who increased their physical activity had a HR 0,41 (0,31-0,55) for all-cause mortality after a mean 4,2 years of follow-up. These patients increased their number of active days from 0,2 to 4,3 days/week on average, which amounts to approximately 14% reduction in mortality risk for each day of increased activity, assuming that the relationship is linear. The difference between attenders and non-attenders in our study was 0.5 days for men and 0.8 days for women, which would amount to 7% and 12% mortality risk reduction, respectively.*

*In Critchley and Capewell´s meta-analysis including 20 studies on smoking cessation post-MI (Mortality risk reduction associated with smoking cessation in patients with coronary heart disease: a systematic review. JAMA. 2003;290(1):86-97) smoking cessation resulted in a 36% relative risk reduction for total mortality. Similar differences have been shown in SWEDEHEART data, as depicted in The SWEDEHEART annual report 2018: (*[*https://www.ucr.uu.se/swedeheart/dokument-sh/arsrapporter-sh/swedeheart-arstrapport-2018/download*](https://www.ucr.uu.se/swedeheart/dokument-sh/arsrapporter-sh/swedeheart-arstrapport-2018/download)*) – p.104.*

*The Discussion has been amended to better acknowledge the above points.*

Without knowing patient physical activity levels at baseline, it is difficult to claim the exercise-based CR increased PA levels.

***Author response:*** *The authors agree. While PA was not assessed at baseline, it was assessed at the first CR follow-up visit approximately 2 months post MI. However, we chose to report the actual values at the second follow-up only, as many of the exCR attenders had started their exCR programme when attending the first follow-up visit and thus potentially biasing the results.*

*We have inserted a new paragraph in the Discussion to acknowledge the Reviewer´s comment.*

Line 261 to 272– This section is quite off topic because the discussion surrounding mortality benefit isn’t focused on the observed significant improvements in cardiovascular risk factors. It would be more valuable to discuss the improvements observed in this trial and how this might improve survival/morbidity. This would be particularly interesting if the changes in cardiovascular risk factors are comparable to those reported in other studies.

***Author response:*** *The discussion has been rewritten, and the above mentioned section has been shortened and moved further back in the text.*

Line 289 – This line relates to the point I highlighted earlier regarding data on how long patients took part in CR for. Please can you discuss this within the methods or results as appropriate.

***Author response:*** *The fact that the number of completed exCR sessions was not documented in the SWEDEHEART registry at the time of the study has been added to the Methods section (Study variables sub-section), and now reads:*

“While it is recommended that the duration of the exercise programme should be at least three months, the number of completed exCR sessions was not documented in SWEDEHEART at the time of the study.”

*The section in the Discussion the Reviewer refers to has also been amended, acknowledging more clearly this limitation to the study data.*

Line 327 – This shouldn’t be a new paragraph if starting with ‘on the other hand’.

***Author response:*** *The sentence has been rephrased.*

## Reviewer #4

This is a really important paper and I have only very minor comments regarding the clarity of difference between exCR and CR in the Patient population settings. Can you please explain how it was possible to say the changes were attributable to exercise and not to the other elements of the comprehensive CR programme?

***Author response:*** *As the reviewer rightfully points out, those attending exCR might have had better attendance in other CR programme elements that have been shown to have beneficial effects on risk factor management and long-term outcomes after CR, such as patient education or nurse-led interventions, causing a potential bias in our findings. Unfortunately data on attendance in other specific elements of the CR programme was not available. This point is highlighted in the Limitations section of the manuscript.*
