# Supplementary material for: Association between attending exercise-based cardiac rehabilitation and cardiovascular risk factors at one-year post myocardial infarction
Source: PLoS One. 2020 May 11;15(5):e0232772. doi: 10.1371/journal.pone.0232772 (PMC7213725; doi:10.1371/journal.pone.0232772)
Supplement: Attachment — Submitted filename: Response to reviewers.docx [file pone.0232772.s002.docx]

20^th^ of April 2020

***Response to reviewers – second revision***

PONE-D-19-32783R1
Association between attending exercise-based cardiac rehabilitation and cardiovascular risk factors at one-year post myocardial infarction

**Reviewer #2**

This is a well written article which helps argue the positive impact of CR upon CVD risk factors (particularly useful given that the effectiveness of CR has been questioned within the literature recently).

There are only minor revisions required:

line 43 - significantly larger than? ('non-attenders' needs added here)

***Author response:*** *The sentence has been rephrased and now reads:*

In an unselected registry cohort of patients post myocardial infarction, compared to non-attenders those attending exercise-based cardiac rehabilitation achieved significantly larger improvements in cardiovascular risk factors at one-year after the acute event.

lines 194-204 - it would be helpful to have the p values after each finding here.

***Author response:*** *p-values have been added to the text.*

line/table 206 - personally I find the placement of the p values confusing, e.g. why is the p value for employment status only aligned with employment status when the text states that that the difference lies between those who are employed and those who aren't? I think the table could be clearer

***Author response:*** *The statistical test used for employment status is chi-square test. As such, the test only says whether there are differences between attenders and non-attenders in sub-group distribution. Placing the p-value after the heading (as opposed to each sub-group) is the preferred way to report chi-square testing with multiple sub-groups. The text in the Results chapter did not fully reflect this, and we thank the author for pointing that out. The text has been amended accordingly, also for smoking status (where the same type of testing was performed).*

lines 285-300 - here it would be good to refer back to reference [8] (as a quite prominent article presenting negative results in relation to CR) and discuss your findings in relation to this.

***Author response:*** *In our opinion the conduction of the meta-analysis by Powell et al was deficient. Firstly, the interventions included in the analysis were heterogenic and included not only moderate-intensity exercise training but also non-supervised walking programmes and text messages intended to influence exercise behaviour. Secondly, in 2/3 of the included studies the follow-up time was ≤1 year, which is in our opinion too short to draw conclusions about mortality, especially in the modern era of CAD treatment. This was evident by the few number of events (0-2 deaths in each group in 68% (total mortality) and 78% (CVD mortality) of the included studies, respectively), limiting conclusions that can be drawn on a lack of effect of exCR on these outcome measures.*

*Finally, Powell only included mortality and hospitalization as outcome measures, not cardiovascular risk factors. Taking the above-mentioned limitations of the paper as well as the differences in outcome measures into consideration we find limited value in discussing their conclusions in relation to our results in the manuscript.*

This is another good reference / notable paper which would be good to discuss in relation to your results:
Van Halewijn G, Deckers J, Tay HY, van Domburg R, Kotseva K & Wood D. Lessons from contemporary trials of cardiovascular prevention and rehabilitation. International Journal of Cardiology 2017; 232:294-303.

***Author response:*** *We agree that the meta-analysis by Van Halewijn et al is a good paper. However, as their analysis included comprehensive CR, i.e. both exercise and lifestyle based programmes, we found it less well suited for inclusion in our discussion. We therefore decided not to include the reference in our paper.*
